# 

**Published:** 1909-03

**Authors:** 


### BOOKS RECEIVED.

Clinical Diagnosis and Treatment of Disorders of the Bladder with Technic of Cystoscopy, by Follen Cabot, M.D., Professor of Genitourinary Diseases, Post-Graduate Medical School; Attending Genitourinary Surgeon, Post-Graduate and City Hospitals, New York. 8 vo, 225 pages. Forty-one illustrations, 1 colored plate. Prepaid $2.00. New York: E. B. Treat & Co., Medical Publishers, 241-243 West 23d street, New York.

Bacterial Food Poisoning. A Concise Exposition of the Etiology, Bacteriology, Pathology, Symptomatology, Prophylaxis, and Treatment of So-Called Ptomaine Poisoning. By Professor Dr. A. Dieudonné, Munich. Authorized translation, edited, with additions, by Dr. Charles Frederick Bolduan, Bacteriologist, Research Laboratory, Department of Health, City of New York. 8vo, 125 pages. New York: E. B. Treat & Co., Medical Publishers, 241-243 West 23d Street. (Cloth, prepaid, $1.00 net.)

The Changing Values of English Speech. By Ralcy Husted Bell. New York: Hinds, Noble & Eldridge, Publishers.

Blood Examination in Surgical Diagnosis. A Practical Study of Its Scope and Technic. By Ira S. Wile, M.D., New York. Duodecimo; 161 pages; 35 illustrations and 1 double-page colored plate. New York: Surgery Publishing Company, 1908. Cloth, price $2.00; oil cloth for laboratory use, $2.50; de luxe ooze leather, price $3.00.

Seven Hundred Surgical Suggestions. Practical Brevities in Surgical Diagnosis and Treatment. By Walter M. Brickner, B.S., M.D., Assistant Adjunct Surgeon, Mount Sinai Hospital, New York; Editor-in-Chief, American Journal of Surgery, Eli Moschcowitz, A.B., M.D., Assistant Physician, Mount Sinai Hospital Dispensary, New York, and Harold M. Hays, M.A., M.D. Third Series. Duodecimo; 153 pages. New York: Surgery Publishing Co., 92 William Street. Price, semi de luxe, $1.00; full library de luxe ooze leather, gold edges, $2.25.

Orthopedic Surgery for Practitioners. By Henry Ling Taylor, M.D. Professor of Orthopedic Surgery and Attending Orthopedic Surgeon, New York Post-Graduate Medical School and Hospital, etc.; assisted by Charles Ogilvy, M.D., Adjunct Professor of Orthopedic Surgery at the same institution; and by Fred H. Albee, M.D., Instructor of Orthopedic Surgery at the same institution. With 254 illustrations. New York and London: D. Appleton & Company. 1909. (Price, $5.00, cloth.)

Transactions of the Fourteenth Annual Meeting of the American Laryngological, Rhinological, and Otological Society, held at Pittsburg, May 28, 29, and 30, 1908. Saint Louis: Published by the Society. 1908.

Hygiea, Medicine and Pharmacy. Monatsskrift Festband. Edited by Professor Dr. Carl Sundberg. Two volumes. Illustrated. Stockholm: Isaac Marcus, Bookseller and Publisher. 1908.

Transactions of the American Gynecological Society. Volume 33. Fourth year, 1908. Edited by J. Riddle Goffe, M.D., Secretary. Philadelphia: William J. Dornan, Printer.

